# On Heisenberg Uncertainty Relationship, Its Extension, and the Quantum Issue of Wave-Particle Duality

**DOI:** 10.3390/ijms11104124

**Published:** 2010-10-22

**Authors:** Mihai V. Putz

**Affiliations:** 1 Laboratory of Computational and Structural Physical Chemistry, Chemistry Department, West University of Timişoara, Pestalozzi Street No.16, Timişoara, RO-300115, Romania; E-Mail: mvputz@cbg.uvt.ro or mv_putz@yahoo.com; Tel.: ++40-256-592-633; Fax: ++40-256-592-620; Web: www.mvputz.iqstorm.ro; 2 Theoretical Physics Institute, Free University Berlin, Arnimallee 14, 14195 Berlin, Germany

**Keywords:** quantum fluctuation, Feynman centroid, de Broglie wave-packet, dispersion relationships, quantum duality

## Abstract

Within the path integral Feynman formulation of quantum mechanics, the fundamental Heisenberg Uncertainty Relationship (HUR) is analyzed in terms of the quantum fluctuation influence on coordinate and momentum estimations. While introducing specific particle and wave representations, as well as their ratio, in quantifying the wave-to-particle quantum information, the basic HUR is recovered in a close analytical manner for a large range of observable particle-wave Copenhagen *duality*, although with the dominant wave manifestation, while registering its progressive modification with the factor $\sqrt{1-n^{2}}$, in terms of magnitude *n*∈**[0,1].** of the quantum fluctuation, for the free quantum evolution around the exact wave-particle *equivalence*. The practical implications of the present particle-to-wave ratio as well as of the free-evolution quantum picture are discussed for experimental implementation, broken symmetry and the electronic localization function.

## 1. Introduction

Since its inception, the Heisenberg Uncertainty Relationship (HUR) [1] has become one of the most fascinating and controversial issue of quantum mechanics. Under its customary presentation

(1) $$\Delta x\Delta p\geq \frac{\hbar }{2}$$

as independently proved by Robertson and Schrodinger [2,3] working out the standard deviation of coordinate (*x*) and momentum (*p*)

(2) $$\Delta x=\sqrt{\langle x^{2}\rangle -{\langle x\rangle }^{2}},\Delta p=\sqrt{\langle p^{2}\rangle -{\langle p\rangle }^{2}}$$

it was eventually criticized as being no more than the experimental realization of the operatorial (non)commutation relation [*x*,*p*] = *iħ* that implicitly contains the incompatibility between the coordinate and momentum spaces [4–6]. It was even claimed that HUR acts like a Copenhagen doctrine propagated in quantum mechanical texts without consistent proof [7], as far as failing to clearly include the quantum fluctuation information for the quantum objects in focus, especially relating with the wave-particle feature [8,9]. As such, no definitive argument was delivered so far in linking HUR with the wave-particle *duality in measurements* (*i.e.*, *when the quantum objects are complementarily manifested either as wave or particle*) nor with the wave-particle *equivalency in free evolution* (that is not necessarily related with free motion but with quantum existence independent of any experiment or observation). Nevertheless, possible generalizations and reformulation of HUR were suggested during the last decade by the modern quantum mathematics [10–12], optics [13], information theory [14–16], still without establishing the HUR description in the absence of commutation rules [17,18] or versions of Schwarz inequality [19,20].

In this context, the actual quest is to present a clear yet effective discussion on how HUR becomes valid without involving any operatorial commutation constraints, through explicitly including the quantum fluctuation, while providing the complementary wave-particle analytical description in which the extended-HUR (E-HUR) is not only possible but necessary.

## 2. HUR by Feynman Periodic Paths

The background of the present approach is the Feynman path integral formulation of quantum mechanics [21]. This is most suitable for our purpose since by its fundament, the path integral approach is a *non-operatorial* formulation of quantum mechanics, *i.e*., operators are simply considered by their working definitions involved in global rather than in local quantum description.

Yet, for being adequate for the measurement conditions the periodic paths have to be considered, *i.e.*, when the final and initial space-points coincide, since only in such a case the particle travels in a very short time not far away from the initial position and then is back to the initial point; such a picture has the physical, measurable consequence that a particle is observed in the initial point, *i.e.*, it is found in a stationary state/orbit, while *the quantum fluctuations* are oscillating around the equilibrium (initial = final) space-point. Analytically, we will consider a quantum statistical periodic path construction *x_a_* = *x*(0) = *x*(*ħβ*) = *x_b_*, with *β* the inverse of the thermal energy *k_B_T* (*k_B_* the Boltzmann constant) for a system in equilibrium at temperature *T* can be constructed by means of the Fourier series

(3) $$x(\tau )=\sum_{m=-\infty }^{+\infty }x_{m}\text{exp}(i\omega_{m}\tau )$$

in terms of the so called Matsubara frequencies *ω_m_*=2*πm/ħβ, m*∈**Z.** Under the condition of real paths, x^*^(τ) = *x*(τ), along the resulted relationship between the coefficients of periodic paths, x^*^_m_=x_−m_=x_m_, the series (3) can be rearranged as the expression

(4) $$x(\tau )=x_{0}+\sum_{m=1}^{+\infty }x_{m}\text{exp}(i\omega_{m}\tau )+c.c.$$

with the 0^th^ term *x*_0_ being known as the Feynman centroid,

(5a) $$x_{0}=\frac{1}{\hbar \beta }\int_{0}^{\hbar \beta }x(\tau )d\tau$$

It represents more than the “zero-oscillating” mode of motion but the thermally averaged path over entire quantum sample [22]:

(5b) $$\begin{array}{c} \frac{1}{\hbar \beta }\int_{0}^{\hbar \beta }x(\tau )d\tau =\frac{1}{\hbar \beta }\int_{0}^{\hbar \beta }\left[x_{0}+\sum_{m=1}^{+\infty }x_{m}\text{exp}(i\omega_{m}\tau )+c.c\right]d\tau \\ =x_{0}+\sum_{m=1}^{+\infty }x_{m}\frac{1}{\hbar \beta }\int_{0}^{\hbar \beta }\text{exp}(i\omega_{m}\tau )d\tau +\sum_{m=1}^{+\infty }x_{-m}\frac{1}{\hbar \beta }\int_{0}^{\hbar \beta }\text{exp}(-i\omega_{m}\tau )d\tau \\ =x_{0}+\frac{1}{\hbar \beta }\sum_{m=1}^{+\infty }x_{m}\int_{0}^{\hbar \beta }[\text{exp}(i\omega_{m}\tau )+\text{exp}(-i\omega_{m}\tau )]d\tau \\ =x_{0}+\frac{2}{\hbar \beta }\sum_{m=1}^{+\infty }x_{m}\int_{0}^{\hbar \beta }\text{cos}(\omega_{m}\tau )d\tau \\ =x_{0}+\frac{2}{\hbar \beta }\sum_{m=1}^{+\infty }\frac{x_{m}}{\omega_{m}}\text{sin}(\omega_{m}\hbar \beta )\\ =x_{0}+\underset{0}{\underset{︸}{\frac{2}{\hbar \beta }\sum_{m=1}^{+\infty }\frac{x_{m}}{\omega_{m}}\underset{0}{\underset{︸}{\text{sin}{\left(2\pi m\right)}_{m\in Z}}}}} \end{array}$$

Being, thus, appropriately interpreted as the average of the observed coordinate at given equilibrium temperature *T*. Remarkably, this way of defining the classical (or observed) *x*_0_ coordinate in terms of averaging of quantum periodic paths (orbits) for a given thermal energy *k_B_T*, stays as an elegant way of relating the classical with quantum nature of an observable (or experiment) without involving the fashioned Fisher information with the rate of entropy increase under Gaussian diffusion condition as a measure of measurement robustness [14].

Instead, here, the philosophy is to introduce appropriately the quantum fluctuation information *a* = *a*(*x*_0_) respecting the average of the observed coordinate (*x*_0_), by the Feynman integration rule founded in the ordinary quantum average (Eqution (6a))

(6a) $${\langle f\rangle }_{a^{2}(x_{0})}=\int_{-\infty }^{+\infty }dx\psi^{*}(x,a^{2}(x_{0}))f\psi (x,a^{2}(x_{0}))$$

for the normalized Gaussian wave-function (Equation (6b))

(6b) $$\psi (x,a^{2}(x_{0}))=\frac{1}{{\left[2\pi a^{2}(x_{0})\right]}^{1/4}}\text{exp}\left[-\frac{{\left(x-x_{0}\right)}^{2}}{4a^{2}(x_{0})}\right]$$

recovering the de Broglie wave-packet [23,24] upon which a quantum property may be estimated.

It is obvious that the Equations (6) fulfill the necessary (natural) condition according which the average of the coordinate over the quantum fluctuations recovers the observed quantity of Equation (5), the Feynman centroid, based on simple Poisson integration rules

(7) $$\begin{array}{c} {\langle x\rangle }_{a^{2}(x_{0})}=\frac{1}{\sqrt{2\pi a^{2}(x_{0})}}\int_{-\infty }^{+\infty }dx[x-x_{0}+x_{0}]\text{exp}\left[-\frac{{\left(x-x_{0}\right)}^{2}}{2a^{2}(x_{0})}\right]\\ =\frac{1}{\sqrt{2\pi a^{2}(x_{0})}}\underset{0}{\underset{︸}{\int_{-\infty }^{+\infty }dx[x-x_{0}]\text{exp}\left[-\frac{{\left(x-x_{0}\right)}^{2}}{2a^{2}(x_{0})}\right]}}+\underset{{\langle x_{0}\rangle }_{a^{2}(x_{0})}}{\underset{︸}{\frac{1}{\sqrt{2\pi a^{2}(x_{0})}}\int_{-\infty }^{+\infty }dx[x_{0}]\text{exp}\left[-\frac{{\left(x-x_{0}\right)}^{2}}{2a^{2}(x_{0})}\right]}}\\ =x_{0}\underset{1}{\underset{︸}{\frac{1}{\sqrt{2\pi a^{2}(x_{0})}}\int_{-\infty }^{+\infty }dx\text{exp}\left[-\frac{{\left(x-x_{0}\right)}^{2}}{2a^{2}(x_{0})}\right]}}=x_{0};\\ {\langle x\rangle }_{a^{2}(x_{0})}={\langle x_{0}\rangle }_{a^{2}(x_{0})}=x_{0} \end{array}$$

The next test is about the validity of the Equation (1)—the HUR itself. To this end the quantities of Equation (2) are computed with the aid of Feynman-de Broglie rule (6); firstly, one gets

(8a) $${\langle {\left(x-x_{0}\right)}^{2}\rangle }_{a^{2}(x_{0})}=\frac{1}{\sqrt{2\pi a^{2}(x_{0})}}\int_{-\infty }^{+\infty }dx{\left(x-x_{0}\right)}^{2}\text{exp}\left[-\frac{{\left(x-x_{0}\right)}^{2}}{2a^{2}(x_{0})}\right]=a^{2}$$

Then, through combining the expression

(8b) $$a^{2}={\langle {\left(x-x_{0}\right)}^{2}\rangle }_{a^{2}(x_{0})}={\langle x^{2}\rangle }_{a^{2}(x_{0})}-2{\langle x\rangle }_{a^{2}(x_{0})}{\langle x_{0}\rangle }_{a^{2}(x_{0})}+{\langle x_{0}^{2}\rangle }_{a^{2}(x_{0})}$$

with the prescription (7) we are left with the actual result

(8c) $${\langle x^{2}\rangle }_{a^{2}(x_{0})}=a^{2}+x_{0}^{2}$$

that, when plugged in the basic Equation (2) alongside the information of >Equation (7), yields the coordinate dispersion

(8d) Δx=a

featuring it in a direct relationship with the quantum fluctuation width.

In the same manner, the evaluations for the integrals of the first and second orders of kinetic moment unfold as

(9a) $$\begin{array}{c} {\langle p\rangle }_{a^{2}(x_{0})}=\frac{1}{\sqrt{2\pi a^{2}(x_{0})}}\int_{-\infty }^{+\infty }dx\text{exp}\left[-\frac{{\left(x-x_{0}\right)}^{2}}{4a^{2}(x_{0})}\right](-i\hbar \partial_{x})\text{exp}\left[-\frac{{\left(x-x_{0}\right)}^{2}}{4a^{2}(x_{0})}\right]\\ =\frac{i\hbar }{2a^{2}(x_{0})}\frac{1}{\sqrt{2\pi a^{2}(x_{0})}}\int_{-\infty }^{+\infty }dx(x-x_{0})\text{exp}\left[-\frac{{\left(x-x_{0}\right)}^{2}}{2a^{2}(x_{0})}\right]=0 \end{array}$$

(9b) $$\begin{array}{c} {\langle p^{2}\rangle }_{a^{2}(x_{0})}=\frac{1}{\sqrt{2\pi a^{2}(x_{0})}}\int_{-\infty }^{+\infty }dx\text{exp}\left[-\frac{{\left(x-x_{0}\right)}^{2}}{4a^{2}(x_{0})}\right](-\hbar^{2}\partial_{x}^{2})\text{exp}\left[-\frac{{\left(x-x_{0}\right)}^{2}}{4a^{2}(x_{0})}\right]\\ =\frac{\hbar^{2}}{a^{2}(x_{0})\sqrt{2\pi a^{2}(x_{0})}}\int_{-\infty }^{+\infty }dx\left[\frac{{\left(x-x_{0}\right)}^{2}}{4a^{2}(x_{0})}-\frac{1}{2}\right]\text{exp}\left[-\frac{{\left(x-x_{0}\right)}^{2}}{2a^{2}(x_{0})}\right]=\frac{\hbar^{2}}{4a^{2}} \end{array}$$

while when plugging them in Equation (2) produce the momentum dispersion expression

(9c) $$\Delta p=\frac{\hbar }{2a}$$

Worth noting is that from the coordinate and momentum dispersions, Equations (8d) and (9c), it appears that the dependency of Planck constant is restricted only to the latter, whereas the quantum fluctuations are in both present, in a direct and inverse manner, respectively.

However, when multiplying the expressions (8d) and (9c) the Heisenberg uncertainty is naturally approached by exact specialization of Equation (1)

$$\Delta x\Delta p=\frac{\hbar }{2}$$

this way resembling in an elegant manner the previous result of statistical complementary observables of position and momentum [15].

For the sake of experimental precision it is worth noting that the error in coordinate localization is given *at least* by one quantum fluctuation “leap” in (8d), *i.e.*, by the width in the de Broglie wave packet of Equation (6) that may be naturally exceeded in certain (large) coordinate observations – from where the general HUR emerges as in Equation (1). Remarkably, the HUR validity was here proved using only the wave-packet properties, including the quantum fluctuation *a* = *a* (*x*_0_) that appears in the final coordinate-momentum multiplied dispersions—being therefore incorporated in the HUR result—a feature not obviously revealed by earlier demonstrations.

Yet, another important idea was raised, namely that the coordinate and momentum dispersions, although in reciprocal relationship with quantum fluctuation, *i.e.*, when during an experiment the quantum fluctuation may be set out in coordinate or momentum it acts larger in the other – and *vice versa*, may be treated somehow *separated*, from where the possibility of different realizations for coordinate dispersion through relations (7) and (8), with consequences for HUR reformulations. Such possibilities and the inter-connection with the wave-particle quantum issue are next explored.

## 3. Extended HUR and the Wave-Particle Quantum Status

We like to identify the general quantum fluctuation conditions in which the HUR is valid and when it is eventually extended. We already note that, whereas the momentum dispersion computation is fixed by relations (9a)–(9c), the evaluation of the coordinate dispersion has more freedom in its internal working machinery, namely:

- considering the condition (7) as an invariant of the measurement theory since it assures the connection between the average over quantum fluctuation of the coordinate and the observed averaged coordinate;
- specializing the quantum (average) relationship (8b) for the condition given by Equation (7);
- obtaining the average of the second order coordinate (8c);
- combining the steps i) and ii) is computing the coordinate dispersion Δ*x* as given by Equation (2).

The present algorithm may be naturally supplemented with the analysis of the wave-particle duality. This is accomplished by means of considering further averages over the quantum fluctuations for the mathematical objects exp(−*ikx*) and exp(−*k*^2^*x*^2^) that are most suited to represent the waves and *particles*, due to their obvious shapes, respectively. Such computations of averages are best performed employing the Fourier *k*-transformation as resulted from the de Broglie packet (6) equivalently rewritten successively as [25]:

(10) $$\begin{array}{c} {\langle f(x,k)\rangle }_{a^{2}(x_{0})}=\frac{1}{\sqrt{2\pi a^{2}(x_{0})}}\int_{-\infty }^{+\infty }dxf[x,k]\text{exp}\left[-\frac{{\left(x-x_{0}\right)}^{2}}{2a^{2}(x_{0})}\right]\\ =\frac{1}{2\pi }\int_{-\infty }^{+\infty }dxf(x,k)\text{exp}\left[-\frac{{\left(x-x_{0}\right)}^{2}}{2a^{2}(x_{0})}\right]\int_{-\infty }^{+\infty }dk^{\prime }\text{exp}\left[-\frac{a^{2}(x_{0})}{2}{k^{\prime }}^{2}\right]\\ =\frac{1}{2\pi }\int_{-\infty }^{+\infty }dxf(x,k)\text{exp}\left[-\frac{{\left(x-x_{0}\right)}^{2}}{2a^{2}(x_{0})}\right]\int_{-\infty }^{+\infty }dk\text{exp}\left[{\left(\frac{x-x_{0}}{\sqrt{2}a(x_{0})}-i\frac{a(x_{0})}{\sqrt{2}}k\right)}^{2}\right]\\ =\int_{-\infty }^{+\infty }\frac{dk}{2\pi }\int_{-\infty }^{+\infty }dxf(x,k)\text{exp}(-ikx)\text{exp}\left[{ikx}_{0}-\frac{1}{2}a^{2}(x_{0})k^{2}\right]\\ =\int_{-\infty }^{+\infty }\frac{dk}{2\pi }f(k)\text{exp}\left[ikx_{0}-\frac{1}{2}a^{2}(x_{0})k^{2}\right] \end{array}$$

With the rule (10) one may describe the average behavior of the wave and particle, respectively as

(11) $$\begin{array}{c} {\langle \text{exp}(-ikx)\rangle }_{a^{2}(x_{0})}=\int_{-\infty }^{+\infty }\frac{dk}{2\pi }\text{exp}\left[-ikx+ikx_{0}-\frac{1}{2}a^{2}(x_{0})k^{2}\right]\\ =\int_{-\infty }^{+\infty }\frac{dk}{2\pi }\text{exp}\left[-ik(x-x_{0})-\frac{1}{2}a^{2}(x_{0})k^{2}\right]\\ =\text{exp}\left[-\frac{{\left(x-x_{0}\right)}^{2}}{2a^{2}(x_{0})}\right]\int_{-\infty }^{+\infty }\frac{dk}{2\pi }\text{exp}\left\{-\frac{a^{2}(x_{0})}{2}{\left[k+i\frac{x-x_{0}}{a^{2}(x_{0})}\right]}^{2}\right\}\\ =\frac{1}{2\pi }\text{exp}\left[-\frac{{\left(x-x_{0}\right)}^{2}}{2a^{2}(x_{0})}\right]\int_{-\infty }^{+\infty }dk^{\prime }\text{exp}\left\{-\frac{a^{2}(x_{0})}{2}{k^{\prime }}^{2}\right\}\\ =\frac{1}{\sqrt{2\pi a^{2}(x_{0})}}\text{exp}\left[-\frac{{\left(x-x_{0}\right)}^{2}}{2a^{2}(x_{0})}\right] \end{array}$$

and

(12) $$\begin{array}{c} {\langle \text{exp}(-k^{2}x^{2})\rangle }_{a^{2}(x_{0})}=\int_{-\infty }^{+\infty }\frac{dk}{2\pi }\text{exp}\left[-k^{2}x^{2}+ikx_{0}-\frac{1}{2}a^{2}(x_{0})k^{2}\right]\\ =\int_{-\infty }^{+\infty }\frac{dk}{2\pi }\text{exp}\left[-k^{2}\left(x^{2}+\frac{a^{2}(x_{0})}{2}\right)+ikx_{0}\right]\\ =\text{exp}\left[-\frac{x_{0}^{2}}{4(x^{2}+a^{2}(x_{0})/2)}\right]\int_{-\infty }^{+\infty }\frac{dk}{2\pi }\text{exp}\left\{-\left(x^{2}+\frac{a^{2}(x_{0})}{2}\right){\left[k-i\frac{x_{0}}{2(x^{2}+a^{2}(x_{0})/2)}\right]}^{2}\right\}\\ =\frac{1}{2\pi }\text{exp}\left[-\frac{x_{0}^{2}}{4(x^{2}+a^{2}(x_{0})/2)}\right]\int_{-\infty }^{+\infty }dk^{\prime }\text{exp}\left\{-\left(x^{2}+\frac{a^{2}(x_{0})}{2}\right){k^{\prime }}^{2}\right\}\\ =\frac{1}{\sqrt{2\pi [2x^{2}+a^{2}(x_{0})]}}\text{exp}\left[-\frac{x_{0}^{2}}{2(2x^{2}+a^{2}(x_{0}))}\right] \end{array}$$

It is worth observing that the practical rule (10) is indeed consistent since recovering in (11) the kernel of the Gaussian de Broglie wave-packet—for the *wave* behavior of a quantum object—as expected. As a consequence, the result (12) may be therefore considered as a viable analytical expression for characterizing the complementary *particle* nature of the quantum manifestation of an object.

Next, the ratio of Equations (11) and (12) is taken

(13) $$\begin{array}{c} \frac{Particle}{Wave}\equiv \frac{{\langle \text{exp}(-k^{2}x^{2})\rangle }_{a^{2}(x_{0})}}{{\langle \text{exp}(-ikx)\rangle }_{a^{2}(x_{0})}}\\ =\sqrt{\frac{a^{2}(x_{0})}{2x^{2}+a^{2}(x_{0})}}\text{exp}\left[-\frac{x_{0}^{2}}{2(2x^{2}+a^{2}(x_{0}))}+\frac{x^{2}-2xx_{0}+x_{0}^{2}}{2a^{2}(x_{0})}\right] \end{array}$$

giving the working tool in estimating the particle-to-wave content for a quantum object by considering various coordinate average information; this will be achieved by (v) making the *formal* identity of the coordinate quantities in Equation (13) with the respective values as furnished by the steps i)–iii) of the above coordinate averages’ algorithm

(14) $$x_{0}↔{\langle x_{0}\rangle }_{a^{2}(x_{0})},x↔{\langle x\rangle }_{a^{2}(x_{0})},x_{0}^{2}↔{\langle x_{0}^{2}\rangle }_{a^{2}(x_{0})},x^{2}↔{\langle x^{2}\rangle }_{a^{2}(x_{0})}$$

since they nevertheless emerge from quantum average operations (measurements).

Now we are ready for presenting the two possible scenarios for quantum evolutions along the associate HUR realization and the wave-particle behavior.

### 3.1. Observed Evolution

For the case of observed quantum evolution, the averaged observed position is considered in relation with the quantum fluctuation by the general relationship

(15a) $${\langle x\rangle }_{a^{2}(x_{0})}={\langle x_{0}\rangle }_{a^{2}(x_{0})}=x_{0}=na,n\in R$$

implying that the average of the second order of Feynman centroid looks like

(15b) $${\langle x_{0}^{2}\rangle }_{a^{2}(x_{0})}=n^{2}a^{2}$$

When (15a) and (15b) are considered into the identity (8c), according with the step iii) above, the actual average of the second order coordinate is obtained

(15c) $${\langle x^{2}\rangle }_{a^{2}(x_{0})}=a^{2}(1+n^{2})$$

Not surprisingly, when further combining relations (15a) and (15c) in computing the coordinate dispersion of Equation (2), *i.e.*, fulfilling the step iv) above, one regains the value of Equation (8d) that recovers at its turn the standard HUR no matter how much the quantum fluctuation is modulated by the factor *n*. However, the P(article)/W(ave) ratio of Equation (13) takes the form

(16) $${\left(\frac{Particle}{Wave}\right)}_{\begin{array}{l} Observed\\ Evolution \end{array}}=\frac{1}{\sqrt{3+{2n}^{2}}}\text{exp}\left(\frac{3+n^{2}}{6+4n^{2}}\right)=\{\begin{array}{l} 0.952\ldots n=0\\ 0.667\ldots n=1\\ 0\ldots n\to \infty \end{array}$$

showing that the wave-particle duality is indeed a reality that can be manifested in various particle-wave (complementary) proportions—yet never reaching the perfect equivalence (the ratio approaching unity). Moreover, because (P/W)_Obs_ < 1, it appears that the general behavior of a quantum object is merely manifested as wave when observed, from which arises the efficacy of spectroscopic methods in assessing the quantum properties of matter.

### 3.2. Free Evolution

Moving to the treatment of the *free quantum evolution*, the average of the first order coordinate is assumed as vanishing

(17a) $${\langle x\rangle }_{a^{2}(x_{0})}={\langle x_{0}\rangle }_{a^{2}(x_{0})}=x_{0}=0$$

since the quantum object, although existing, is not observed (see the spontaneous broken symmetry mechanism in the Discussion Section 4 below).

The relation with quantum fluctuation may be nevertheless gained by the average of the second order of the Feynman centroid–considered under the form

(17b) $${\langle x_{0}^{2}\rangle }_{a^{2}(x_{0})}=n^{2}a^{2}$$

Note that Equations (17a) and (17b) parallel the statistical behavior of error in measurements that being vanishing in the first case as mean deviation, is manifested in the second as squared deviation (dispersion), respectively.

Next, through recalling the referential Equation (8b)—the step ii) in above algorithm—the average of the second order coordinate provides now the expression

(17c) $${\langle x^{2}\rangle }_{a^{2}(x_{0})}=a^{2}(1-n^{2})$$

The result (17c) restrains the domain of the free evolution quantum fluctuation factor *n* to the realm *n*∈**[0,1].** With Equations (17a) and (17c), the step iii) in above algorithm, one finds the coordinate dispersion

(18) $$\Delta x=a\sqrt{1-n^{2}}$$

with the immediate consequence in adjusting the basic HUR as

(19) $$\Delta x\Delta p\geq \frac{\hbar }{2}\sqrt{1-n^{2}}$$

On the other hand, within conditions fixed by Equations (17a) to (17c) the P(article)/W(ave) index of Equation (13) becomes

(20) $${\left(\frac{Particle}{Wave}\right)}_{\begin{array}{l} Free\\ Evolution \end{array}}=\frac{1}{\sqrt{3-2n^{2}}}\text{exp}\left(\frac{3-n^{2}}{6-4n^{2}}\right)=\{\begin{array}{l} 0.952\ldots n=0\\ 1\ldots n_{\Omega }=0.54909\\ 1.048\ldots n=0.87\\ 1\ldots n_{\alpha }=1 \end{array}$$

Through characterizing the numerical results of Equation (20), one firstly observes that they practically start from where the P/W function of Equation (16) approaches its highest output. In other words, this tell us remarkable information according to which the *observed and free quantum evolutions are continuous realities, being smoothly accorded in the point of precise measurement* (*n* = 0). Another very interesting observation is that the P/W ratio symmetrically spans in (20) the existence domain either for wave P/W∈ [0.952, 1) or particle P/W∈ (1, 1.048] manifestations around their exact equivalence P/W = 1. However, the precise wave-particle equivalence is two-fold, namely in the so-called *omega* (Ω) and *alpha* (α) points of Equation (20) characterized by the extended HUR versions of Equation (19); written, respectively, as

(21) $${\left(\Delta x\Delta p\right)}_{\Omega }\geq 0.418\hbar$$

(22) $${\left(\Delta x\Delta p\right)}_{\alpha }\geq 0$$

It is clear that whereas the omega case of Equation (21) is characterized by the restrained quantum domain of ordinary HUR of Equation (1), in which a quantum object’s evolution may be grated, on the alpha point of Equation (22) any quantum information is lost since no Planck constant exists there to drive the wave-particle quantum inter-conversion. It is this last case that may be eventually related with early cosmological stages when the quantum fields and particles are considered as absorbed in the universal gravity; nevertheless, this is just a hint for future possible use of the present extended-HUR phenomenology that may help in understanding the occurrence of the quantum information, entanglement, and the separation of the fields and particles towards the observed world.

## 4. Discussion

**Figure 1:**
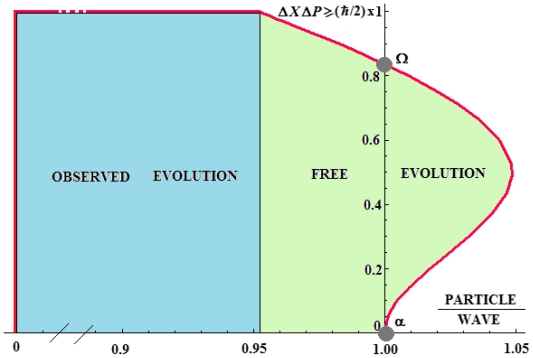
The chart of Heisenberg Uncertainty Relationship (HUR) appearance for observed and free quantum evolutions covering the complete scale of the particle to wave ratios as computed from the Equations (16) and (20), respectively; the points Ω and α correspond to wave-particle precise equivalence and to the special extended-HURs of Equations (21) and (22), respectively.

It is very instructive to present in a unitary manner the observed and free quantum evolution cases in the chart of Figure 1 by linking the HUR shapes of Equations (1) and (19) with the particle/wave ratios values of Equations (16) and (20), respectively. The P/W contribution spreads from the exclusively undulatory quantum manifestation (P/W = 0) in the *observed* domain of quantum evolution until the particle dominance (P/W > 1) in the *free* domain of quantum evolution.

Note that the possibility a quantum object is manifested *only* under particle behavior (*i.e.*, for P/W→∞) is forbidden; this is an important consequence of the present analytical discourse that is in agreement with the Copenhagen interpretation according which the quantum phenomena are merely manifested as undulatory (*viz.* Schrödinger equation) although some particle information may be contained but *never* in an exclusive manner (naturally, otherwise the Newtonian object would exist with no Planck constant and HUR relevance upon it).

However, the wave-particle *duality* matches perfectly and always with HUR in its standard (Schrödinger) formulation of Equation (1); on the other side, the wave-particle exact equivalence (P/W = 1) may be acquired only in the free evolution regime that, in turn, it is driven by modified HUR as given by Equation (21). In other words, it seems that any experiment or observation upon a quantum object or system would destroy the P/W balance specific for free quantum evolution towards the undulatory manifestation through measurement.

Yet, having the analytical expressions for both observed and free quantum evolutions may considerably refine our understanding of macro- and micro-universe. For instance, with various (P/W)_Observed_, one can evaluate the appropriate particle-to-wave presence in a quantum complex for which experimental data are available: once knowing from a given measurement the quantities 〈 *x*_0_^2^〉*_Exp_* and 〈 *x*^2^〉*_Exp_,* with *x*_0_ and *x* appropriately considered for each type of experiment (e.g., the statistical mean for classical records and the instantaneous values for quantum measurement of coordinate, respectively), one can employ Equations (15b) and (15c) to find the magnitude of the quantum fluctuation

(23) $$n=\sqrt{\frac{{\langle x_{0}^{2}\rangle }_{Exp}}{|{\langle x^{2}\rangle }_{Exp}-{\langle x_{0}^{2}\rangle }_{Exp}|}}$$

that when replaced into Equation (16) predicts the P/W ratio involved in that observation.

It is worth giving a working example for emphasizing the reliability of the present approach and to choose for this aim the fundamental Compton quantum experiment. In this case, the incoming photonic beam carries the wavelength λ_0_ whilst the scattered one departs from that incident with the amount Δλ = λ – λ_0_; such situation allows the immediate specialization of the quantum fluctuation magnitude (23) to its Compton form

(24) $$n_{Compton}=\sqrt{\frac{\lambda_{0}^{2}}{(\lambda +\lambda_{0})\Delta \lambda }}$$

Now we can interpret the various experimental situations encountered, employing the output of Equation (24) to asses through Equation (16) the wave-particle ratio degree present in specific measurements. For example, when the scattering is made on *free electrons*, then the higher and higher record for Δ*λ* implies the decrease of *n_Compton_* of Equation (24) and consequently the increase of (P/W)*_Compton_* of Equation (16); this is in accordance with the fact that *the scattered light on free electrons rises more and more its particle (photonic) behavior*. On the other side, when the scattering is made on *tight bonded electrons* (e.g., electrons in atoms of a material), the Compton wavelength departure is negligible, Δλ→0, leaving from Equation (24) with the asymptotic higher quantum fluctuation magnitude *n_Compton_* → ∞ that corresponds at its turn with (P/W)*_Compton_* = 0 in Equation (16). This matches with the fact that this case corresponds with *complete wave manifestation of light that scatters bonded electrons*, resembling the (classical) interpretation according which the scattered bounded electron by a wave entering in resonance with it while oscillating with the same frequency. Therefore, the reliability of the present (P/W)_Observed_ formalism was paradigmatically illustrated, easily applied to other quantum experiments, while giving the numerical P/W estimations once having particular data at hand. Equally valuable is the free evolution (P/W)_Free_ ratio of Equation (20) that may be employed for the wave-particle equivalency between the quantities (11) and (12)

(25) $${\langle \text{exp}(-ikx)\rangle }_{a^{2}(x_{0})}≅{\langle \text{exp}(-k^{2}x^{2})\rangle }_{a^{2}(x_{0})}$$

with an important role in assessing the stability of matter, from atom to molecule. As an example, the justification of the Hydrogen stability was successfully proved through setting the ratio P/W = 1 in the omega point of function (20) or within its vicinity [25,26]. Nevertheless, further applications of the (P/W)_Free_ function (20) and of subsequent modified HUR may be explored also in modeling the various stages and parts of the Universe that cannot be directly observed, as well as when dealing with quantum hidden information in the sub-quantum or coherent states [27,28].

On the other side, one would wish to further discuss the free quantum *vs.* observed quantum evolutions in terms of simple average of paths, *viz.* Equations (17a) and (15a), with practical examples, respectively. The best paradigm that can transform the first into the last one stands the *spontaneous symmetry breaking* [29] that has the role in turning the intrinsic zero ensemble averages of Equation (17a) to the finite observable quantum effects (and fluctuations) of Equation (15a). The best examples are the magnetization and the condensation phenomena: in the first case, due to the invariance under rotation of the Hamiltonian, the ensemble average of the total magnetic moment ***M*** is always zero, <***M*** >= 0, since *+****M*** and −***M*** occur with the same probability [30]. In the case of condensation (for instance Bose-Einstein), the order parameter 〈*ψ*〉 that is obtained from averaging the bosonic fields on the canonical ensemble gives zero result in free (untouched) evolution, 〈*ψ*〉 = 0, due to the inner annihilation nature of the bosonic field 〈*ψ*〉, beside the total Hamiltonian is global gauge invariant under the transformation *ψ*(*x*) →*e^iθ^* *ψ*(*x*), ∀ *θ* ∈ℜ that corresponds with the conservation of the total number of particles in the system [31]. However, either case is resolved within experiments by simple observation (e.g., the ferromagnets and the superfluid ^4^He appear under natural conditions without special experimental conditions) through the so–called “Goldstone excitations” (spin waves and the phonons for ferromagnets and superfluids, respectively) that eventually turns (brakes) the microscopic (free evolution) Hamiltonian symmetry into the macroscopic (observed or directional evolution) symmetry. This mechanism of broken symmetry fits with the present free-to-observed quantum evolution picture since, when revealed, it involves a countless number of zero-energy (yet orthogonal) ground states, leading with the rising of the locally (Goldstone) excited state from one of the ground states that gradually changes over the space from the zero energy and infinity wavelength to some finite non-zero energy and long wavelength; such behavior parallels the turning of the condition of Equation (17a) into that of Equation (15a), where the exact Heisenberg principle is obeyed—however in different Particle/Wave ratios (depending on the phenomenon and experiment), see the above discussion and the Figure 1.

For advanced molecular physical chemistry, it is worth pointing out that the particle/wave ratio (P/W) of Equation (13) may be used to re-shape the so-called *electronic localization function* (ELF) [32], which carries much information on the electronic probability to be manifested as wave or particle in chemical bonding [25,33]. As such, further identification of ELF with the quantity of P/W in the observed regime of Equation (16)

(26) $${ELF}_{P/W}={\left(\frac{Particle}{Wave}\right)}_{\begin{array}{l} Observed\\ Evolution \end{array}}\leq 0.95$$

tells us that, in accordance with the recent interpretation of ELF as error in electronic localization [34], the maximum prescribed error of localization of electrons in atoms and molecules is limited within the range [0,0.95] and can never be complete; *i.e.*, the electron is localizable at least as 5% from its particle contents. In other words, the present approach prescribes that any chemical bond contains at least 5% of particle nature of its pairing electrons, *i.e.*, the covalence is never complete while always coexisting with some ionicity! This is a fundamental result of actual exact HUR treatment for chemical bonding. However, further application of the ELF_P/W_ index (26) for explaining—for instance—the molecular aromaticity [35] in terms of geometry of bonding and the amount of quantum fluctuation present, are in progress and will be in the future communicated.

Finally, for spectroscopic analysis, one could ask upon the corresponding time-energy uncertainty relationship [36] within the actual approach. Firstly, the correctness of such problem is conceptually guaranteed by the Heisenberg representation of a quantum evolution, where, for a cyclic vector of state (*viz.* the present periodical paths or orbits) and an unitary transformation *U*, the cyclic Hamiltonian *H_U_* is accompanied by the time operator *t_U_*= − i*ħ*∂*_μ_* with the ∂*_μ_*= *d*/*d_μ_*(*ɛ*) relating the integrable measure *μ*(*ɛ*) as depending of the energetic spectra (*ɛ*) on the associate generalized Hilbert space [37]. On the other side, quantitatively, the time-energy HUR faces with the practical problem in evaluating the general yield of the Hamiltonian variance

(27a) $$\Delta H=\sqrt{\langle H^{2}\rangle -{\langle H\rangle }^{2}}$$

since containing the non-specified external potential dependency:

(27b) $$H=-\frac{\hbar^{2}}{2M}\partial_{x}+V(x)$$

Yet, the present periodic path approach may be eventually employed to assess the problem through reconsidering the width *a*(*x*_0_) of the de Broglie wave-function (6b) as related with the averaged potential over the quantum fluctuations 〈*V*(*x*)〉*_a_*_^2^(_*_x_*__0_)_; a self-consistent equation is this way expected, while the final time-energy HUR may further depend on the ground or excited (Wigner) states considered, *i.e.*, within the inverse of the thermal energy limits *β* → ∞ or *β* → 0, respectively. Nevertheless, this remains a challenging subject that will be also approached in the near future.

## 5. Conclusion

It is widely recognized that despite the huge success of quantum mechanics, since forecasting the experimental observations, its basic conceptual consequences, namely the *wave-particle duality* and the *uncertainty* issues, have resisted so far any severing of the analytical inter-connection due to the absence of a clear description on how the quantum fluctuation enters the particle and wave quantum manifestations.

The present endeavor made such a step towards providing a unified answer on these fundamental quantum problems by the aid of the Feynman periodic path methodology adapted to compute the coordinate and momentum standard deviations in terms of the quantum fluctuation and the averages of the observed coordinate (the Feynman centroid).

The approach successfully resembles the basic Heisenberg uncertainty relationship (HUR) by showing the reciprocal quantum fluctuation contributions in coordinate and momentum dispersions, yet without employing any operatorial identity or commutation rule. However, the present HUR proof emphasizes the correct role the quantum fluctuation rather than the Planck constant has in uncertainty, it being directly related with coordinate and inversely correlated with momentum uncertainties in measurements.

Moreover, the wave-particle quantum issue was adequately unfolded as well by assessing two types of quantum fluctuation contributions to the first and second orders of coordinate averages. This way, it was found that the wave-particle complex covers two continuously connected realities: one observed and the other of free evolution, yet each of them being analytically characterized by a specific P(article)/W(ave) ratio function.

We found that while the observed reality is fully covered by the standard HUR albeit with an undulatory predominant manifestation of the quantum objects, P/W ∈ [0, 0.952], the free evolution corresponds with isolated (not measured) quantum systems/states with a symmetrical appearance between the particle and wave dominant manifestations around their perfect equivalency, P/W ∈ [0.952, 1.048], however, with the price of altering HUR realization with the factor $\sqrt{1-n^{2}}$ in terms of the quantum fluctuation magnitude *n*∈**[0,1].**

Overall, the present work offers strong analytical arguments in favor of Copenhagen interpretation (consecrated either by the Bohr’s complementarity or by the de Broglie pilot-wave/double-solution pictures) [24] according to which, when observed, the quantum objects are rather manifested as waves than as particles over the quantum fluctuations of concerned systems, in an inextricable connection with the consecrated Heisenberg uncertainty that is altered only in the free evolution regime.

On the other side, the ever residual particle manifestation in whatever system that accompany the wave character of quantum observed evolutions, further allows characterization of the chemical bond by the covalent-ionic mixture as an important molecular specialization of the wave-particle quantum physical paradigm; moreover, the particle-to-wave ratio may provide a working electronic localization function to be further used in understanding bonding properties in direct relation with molecular data assay through the recorded information and computed quantum fluctuation magnitude: see Equation (23).

However, through the Heisenberg uncertainty it is hopefully better integrated in the quantum “measurement dogma” herewith, the numerical predictions of the wave-particle character for both experimental and theoretical approaches are advanced within the reunited {observed ∪ free} evolutions of the quantum objects, by means of the associate P/W functions depending only on the quantum fluctuation magnitude factor rather than on other statistical information.
